# Cardioprotection of Electroacupuncture for Enhanced Recovery after Surgery on Patients Undergoing Heart Valve Replacement with Cardiopulmonary Bypass: A Randomized Control Clinical Trial

**DOI:** 10.1155/2017/6243630

**Published:** 2017-02-16

**Authors:** Fangxiang Zhang, Xiangdi Yu, Hong Xiao

**Affiliations:** Department of Anesthesiology, Guizhou Provincial People's Hospital, Guiyang, China

## Abstract

We attempted to investigate cardioprotection of electroacupuncture (EA) for enhanced recovery after surgery on patients undergoing heart valve replacement with cardiopulmonary bypass. Forty-four patients with acquired heart valve replacement were randomly allocated to the EA group or the control group. Patients in the EA group received EA stimulus at bilateral Neiguan (PC6), Ximen (PC4), Shenting (GV24), and Baihui (GV20) acupoints twenty minutes before anesthesia induction to the end of surgery. The primary end point was cardioprotection effect of electroacupuncture postoperatively and the secondary endpoints were quality of recovery and cognitive functioning postoperatively. The present study demonstrated that electroacupuncture reduced the occurrence of complications and played a role of cardioprotective effect on patients after heart valve replacement surgery with cardiopulmonary bypass, and it benefits patients more comfortable and contributes to recovery after surgery. This trial is registered with ChiCTR-IOC-16009123.

## 1. Introduction

It is well known that cardiopulmonary bypass and surgical procedure contribute to the myocardial ischemia-reperfusion and are associated with high morbidity and mortality after open heart surgery [1]. AS is reported [2, 3], the morbidity of cardiovascular adverse events after coronary artery bypass graft (CABG) reached 27.7% in the first year and early postoperation complications with heart valve replacement occurs in 26.6%, including valve-related events (7.0%), arrhythmia (7.8%), and general complications (11.8%). Severe pain and PONV often due to secondly complications such as pulmonary atelectasis, infection, disorder of bowl function, and prolong to the length of hospital. There remains, however, potentially a lack of clinical application, of enhanced recovery techniques in open heart patients; therefore, novel interventions are indicated to improve clinical outcomes.

In China, acupuncture has been used to treat a wide variety of diseases. Its major advantages are that it is minimally invasive, safe for high-risk patients, and economic with a low risk of complications. EA is a technique similar to the regular acupuncture, which is conducted by inserting acupuncture needles into acupoints and then changing electric stimulation parameters, including the stimulation frequency, current intensity, pulse width, and pulse interval [4]. Compared with regular acupuncture, the acupoint of EA is not as precise as regular acupuncture, since current delivered by needles stimulates a larger area than that of needle itself. Protective effect of EA against myocardial I/R injury in rats has been verified in recent years [5, 6]. Meanwhile, some have reported the cardioprotection of electroacupuncture in the clinic study but not adequate enough [7, 8]. According to the theory of traditional Chinese medicine, the Neiguan (PC6) and Ximen (PC4) acupoints, which belongs to the hand-jueyin pericardium meridian, which is considered to exert a cardioprotective effect, were reported to decrease myocardial ischemia in patients with heart diseases [5, 7]. Otherwise, PC6 is usually a key acupuncture point for preventing and reducing the incidence of PONV, perioperative analgesic requirements, and postoperative pain [9–11]. According to modern medicine, in the projection area of the motor and sensory cortex, as well as in the projection area of the anterior cerebral artery which is Baihui acupoint (GV20), it is probably an important acupoint in preventing and treating cerebral diseases, and it enhances the adults' postoperative cognitive function [12–16]. Shenting (GV24) is a useful acupoint with effects of sedation and treating dysphoria [17]. Whether EA is beneficial for patients' enhanced recovery after cardiovascular surgery is not clear. Therefore, in this paper, a randomized controlled clinical trial was designed to assess the cardioprotective effects of EA at Neiguan (PC6), Ximen (PC4), Shenting (GV24), and Baihui (GV20) acupoints on patients contributing to enhanced recovery after heart valve replacement surgery.

## 2. Methods

The study protocol was approved by the ethics committee of Guizhou Provincial People's Hospital and was performed according to the Declaration of Helsinki (1996) and all relevant Chinese laws. The trial was registered at http://www.chictr.org.cn/ (ChiCTR-IOC-16009123) Written informed content was obtained from all patients before inclusion.

### 2.1. Patients

From December 2015 to March 2016, 148 patients were diagnosed with rheumatic heart disease with severe valve impairment and for whom the clinical decision had been made to perform valve replacement operation. Patients with age range from 18 to 55 years, cardiac functional class III (New York Heart Association), ASA (American Society of Anesthesiology) state III, and BMI 18~25 kg/m^2^ were eligible for enrollment. Excluded criteria were as follows: patients with the comorbidities of coronary heart disease, hypertension, diabetes mellitus, acute or chronic pneumonia, cerebral infarction, severe heart failure, and arrhythmia or who were undergoing reoperation and operation combined with radiofrequency ablation and removal of a blood clot.

### 2.2. Randomization and Blinding

**Figure 1:**
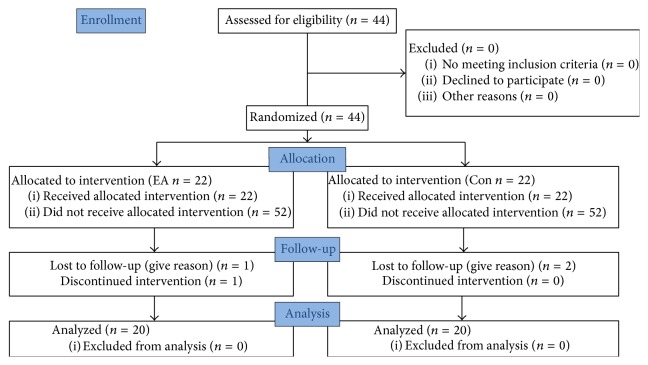
Consort flowchart.

Patients were assigned to either EA stimulus (EA group) or control group (Con group) on the basis of random numbers generated by a computer before the start of surgery by the nurse. Only the acupuncturist was informed the randomization allocation by the nurse, just before the onset of EA. None of the anesthesiologists, surgeons, and physicians in the postanesthesia care unit (PACU) or participants were aware of the allocation (Figure 1).

### 2.3. Electroacupuncture Technique

**Figure 2:**
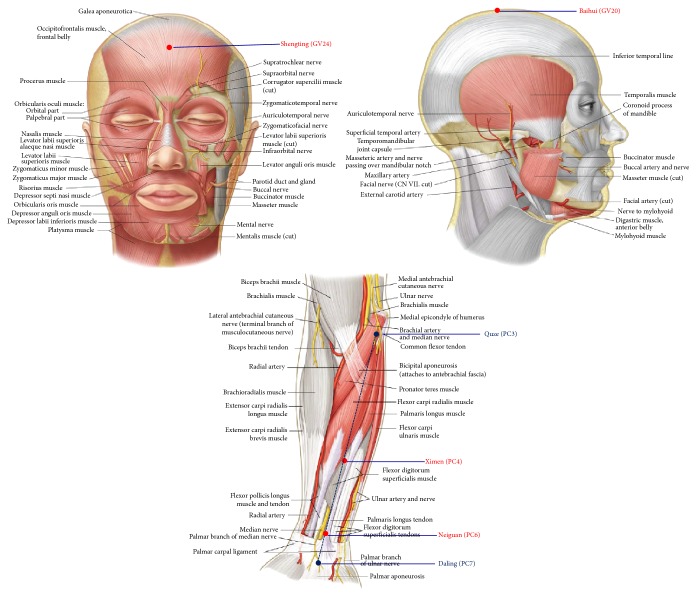
Location of acupoints. Baihui (GV20), located on the continuation of the line connecting the lowest and highest points of the ear, on the median line of the head, 7 cun above the posterior hairline, and 5 cun behind the anterior hairline; Neiguan (PC6), on the palm side of the forearm and on the line connecting Quze (PC3) and Daling (PC7), 2 cun above the crease of the wrist; Xinmen (PC4), on the palm side of the palm side of forearm and on the line connecting Quze (PC3) and Daling (PC7), 5 cun above the crease of the wrist; Shenting (GV24), on the median line of the head, 0.5 cun directly above the midpoint of the anterior hairline.

An experienced acupuncturist performed EA stimulus before twenty minutes of anesthesia induction to the end of surgery. For the patients in the EA group, acupoints chosen were at bilateral heart meridian of Neiguan (PC6), Ximen (PC4), Shenting (GV24), and Baihui (GV20) acupoints whose position is the across of hundred meridian (Figure 2). The stainless steel needle with diameter of 0.3 × 25 mm was connected to SDZ-IV Electronic Acupuncture Treatment Instrument (Chinese Medicine Apparatus, Jiangsu, China); the puncture is made in accordance with the standards of TCM, to the depth of 1 to 3 cm depending on the thickness of the local tissues. The EA parameter was set at density-sparse wave with the frequency of 2 HZ/100 HZ at a current of the patients' tolerance best ranged from 0.5 mA to 1.2 mA.

Patients randomized to the Con group receive sham EA treatment. For sham EA, the short stainless steel needle is inserted to a depth of 1 to 2 mm, which is deep enough to make the needle stand up on the skin. The needles are only connected to the EA device without any current passing through. Other treatment procedures are similar to those of the EA group.

### 2.4. Anesthesia and Cardiopulmonary Bypass

One surgeon conducted all surgeries according to a standard protocol; surgery commenced between 8:30 and 1:00 p.m. Anesthesia was induced with midazolam 0.1 mg/kg, etomidate 0.3 mg/kg, vecuronium 0.1 mg/kg, and sufentanil 0.5 *μ*g/kg. The patients were mechanically ventilated after tracheal intubation. Anesthesia was maintained with inhalation of 1%~2% sevoflurane, propofol 4~6 mg/kg/h, and sufentanil 0.3~0.8 *μ*g/kg/h intravenous infusion and intermittent i.v. infusion of vecuronium. Standard monitoring was used, including a radial or femoral artery catheter for measurement of systemic arterial blood pressure, internal jugular vein catheter for measurement of central venous pressure, and accumulating blood samples.

The extracorporeal circuit was primed with balanced acetate solution, whole blood, and Plasma-Lyte A pH 7.4 (Multiple Electrolytes Injection, Type 1, USP), with the aim of obtaining a hematocrit of 20%~25% at the time of onset of low-flow CPB. A nonpulsatile roller pump with a membrane oxygenator (Medtronic. Inc., Minneapolis, USA) was used, and flows of 1.8 to 2.2 L/(min·m^2^) were obtained with a roller pump under low hypothermia (29°C to 31°C).

After discontinuation of CPB, anticoagulation was reversed with protamine sulfate. Blood remaining in the CPB circuit was collected and infused to the patient before transfer to the intensive care unit (ICU).

### 2.5. Measurements of Serum Samples

Plasma concentrations of cTnI and hFABP were planned to measure. For each patient, 5 ml blood samples were taken at six time points: (T0) 30 min before electroacupuncture, 30 min of CPB (T1), 30 min (T2), 60 min (T3), and 120 min (T4) after CPB and 360 min (T5) and 1440 min (T6) after operation, of which 0.3 ml was used to blood gas analysis for obtaining Hct. The blood was transferred into dry glass tubes and stored at 4° to 8° before centrifugation. Plasma separated after centrifugation was frozen at −70° until being assayed. The plasma samples were measured in the central laboratory of our hospital by individuals unaware of the group allocation.

ELISA was used to detect plasma concentrations of cTnI and hFABP. The ELISA-kit was purchased from Boyun Biotech Company (Shanghai Boyun Biotech Co. Ltd., China). The standard curve was generated using the reference standard set supplied in the kit. The samples were measured according to the instructions accompanying the kit. The results were read using a microplate reader (Elx-800, Bio-Tek instruments, Inc., USA) at wavelengths of 450 nm.

### 2.6. Arrhythmia Scoring

The arrhythmia scoring system was adopted to assess arrhythmia score for 24 h after operation [18, 19]. The principles of the scoring system employed were as follows. (1) Ventricular arrhythmias are more severe than atrial arrhythmias; (2) the severity of ventricular arrhythmias is ventricular fibrillation (VF), ventricular tachycardia (VT), frequent premature ventricular contraction (PVC), and occasional PVC in descending order; (3) the longer the duration of arrhythmias or the more frequent the incidence of arrhythmias, the greater the severity of arrhythmias. In the present study, the score of a heart was that of the most severe type of arrhythmia exhibited by the heart. The details of the scoring system are shown in the Table 1.

### 2.7. Quality of Recovery-9 (QoR-9) and Minimum Mental State Examination (MMSE)

Chinese versions of QoR-9 and MMSE were translated from English version by a medical English expert.

The Quality of Recovery-9 (QoR-9) is a validated scale with 5 domains [20–22]. These measure physical comfort, emotional state, physical independence, psychological support, and pain. Each domain is scored to a maximum global score of 200. QoR-9 scores have been found to be associated with both quality-of-life scales and patient satisfaction indices [23] as well as postoperative pain [24]. In the present study, QoR-9 evaluation was performed on preoperative day 1 (D0), postoperative day 1 (D1), and postoperative day 3 (D2).

The Mini-Mental State Examination (MMSE) is one of the most widely used assessment instruments of cognitive functioning postoperatively, which screens domains of orientation to time and place, attention and memory, concentration, language, and praxis [25]. Patient cognitive function was assessed using the MMSE on preoperative day 1 (D0), postoperative day 1 (D1), and postoperative day 3 (D2).

### 2.8. Data Collection

Perioperation and postoperative data for the study population were collected prospectively by the study team from the day of surgery until hospital discharge. The perioperative data included age, sex, weight, heart function (NYHA) degree, left ventricular ejection fraction (LVEF), and rhythm of heart (sinus or atrial fibrillation) before surgery. The operative data included valve replace type, CPB time, aorta cross-clamp time, anesthesia time, the consumption of anesthetics, operation time, and type of rhythm recovery (spontaneous or electric defibrillation).

The postoperative data include the extubation time, first flatus time, the length of stay in the ICU, and length of posthospital stay. All the complications were recorded especially PONV and POCD.

### 2.9. Statistical Analysis

The data were recorded in an Excel spreadsheet and analyzed using SPSS statistical software (version 17; SPSS Inc., Chicago, IL, USA). All the data were presented as mean ± SD. Repeated measures analysis of variance (ANOVA) was used to compare differences at different time points in one group and Mauchly's test was used to determine sphericity. Two-group comparison was determined using unpaired 2-tailed Student's *t*-test. Categorical variables were reported as the number of patients (%) and evaluated using the *χ*^2^ test. The threshold for significance was set at *P* < 0.05.

## 3. Results

Of 148 patients, 44 patients met the inclusion criteria and were randomized to control group and EA group, and 40 patients completed the study. Four patients were excluded because of using temporary cardiac pacemaker after surgery (3) or giving up to assess (1), forty patients records were analyzed, and electroacupuncture showed no side effects at no additional patient cost.

### 3.1. Patient Characteristics

The basic characteristic of study population revealed no significant differences between groups regarding gender, age, weight, LVEF, and valve replacement or heart function degree and rhythm of heart before surgery. Groups were comparable with regard to anesthesia time, operation time or CPB time, aorta cross-clamp time, and total volume (*P* > 0.05), which indicated that the randomized allocation of the patients into two study groups was successful. Though anesthetics consumption between two groups including midazolam, vecuronium, and propofol made no significance, the dosage of sufentanil given in EA group was less than control group (Table 2).

### 3.2. Data Collection of Recovery after Surgery

Time to extubation, first flatus time, first out of bed activity, ICU retention time, and the length of posthospital stay after operation were all significantly shorter in the EA group (Table 3). The incidence of PONV and POCD was significantly reduced in the EA group (Table 3).

### 3.3. MMSE Scores and QoR-9 Scores (Secondary Outcomes)

**Figure 3:**
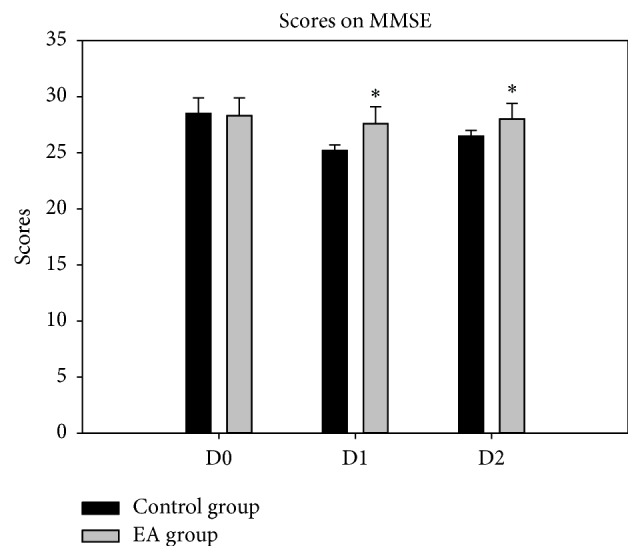
Scores on MMSE. ^*∗*^*P* < 0.05* versus* control group.

**Figure 4:**
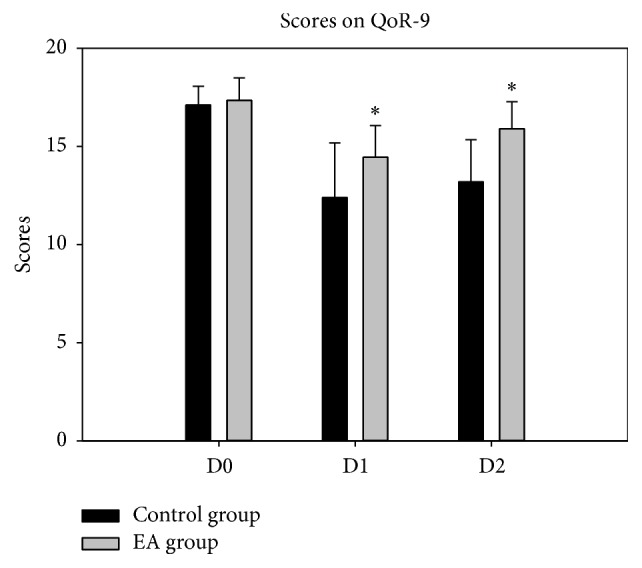
Scores on QoR-9. ^*∗*^*P* < 0.05* versus* control group.

QoR-9 scores of D0 showed no difference between two groups; compared with control group, QoR-40 scores of D1 and D2 were higher in EA group (14.5 ± 1.6 versus 12.8 ± 2.2; 15.9 ± 1.4 versus 13.2 ± 2.1, resp.). MMSE scores of D0 and D2 showed no difference between two groups; compared with control group, MMSE scores of D1 were significantly higher to EA group (27.6 ± 1.5 versus 26.2 ± 0.5); they were all lower after surgery than before in MMSE scores and QOR-9 scores in the two groups (Figures 3 and 4).

### 3.4. The Effects of Cardioprotection (Primary Outcomes)

We noticed no significant differences in the type of heartbeat restored after aorta cross-clamp removal whatever spontaneous or defibrillation; though the total occurrence of arrhythmia after surgery showed no difference between the two groups, rapid ventricular arrhythmias were markedly reduced in the EA group comparison to the control group in the first three days, and arrhythmia score in the EA group was 1.2 ± 0.3 and significantly lower to the control group (Table 4).

**Figure 5:**
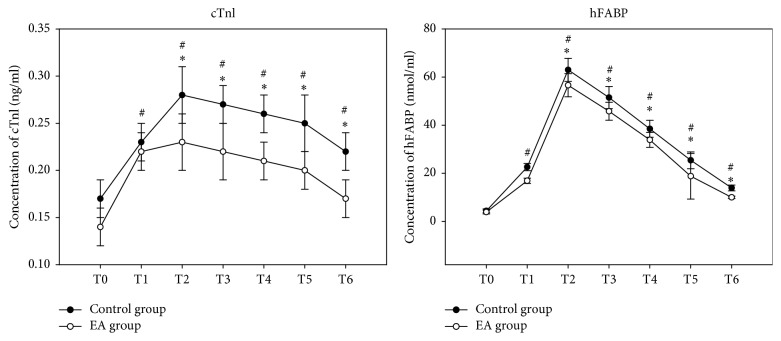
Serum concentrations of mediators of cardiac injury. ^#^*P* < 0.05* versus* T0; ^*∗*^*P* < 0.05* versus* control group.

As shown in Figure 5, there were no significant differences before surgery of cTnI between the two groups. The serum concentration of cTnI from T1 to T6 was rapidly increased compared to that of before surgery (Con group: T1* versus* T0 *P* = 0.012, T2* versus* T0 *P* < 0.001, T3* versus* T0 *P* = 0.002, T4* versus* T0 *P* = 0.005, T5* versus* T0 *P* = 0.0007, and T6* versus* T0 *P* = 0.016; EA group: T1* versus* T0 *P* = 0.018, T2* versus* T0 *P* = 0.011, T3* versus* T0 *P* = 0.013, T4* versus* T0 *P* = 0.015, T5* versus* T0 *P* = 0.027, and T6* versus* T0 *P* = 0.036) and reached the peak point at T2; however, compared to control group, the serum concentration of cTnI from T2 to T6 was significantly less in EA group (EA group* versus* Con group: T2, *P* = 0.046; T3, *P* = 0.021; T4, *P* = 0.019; T5, *P* = 0.010; T6, *P* = 0.009). Interestingly, all patients showed a significant increase in the serum concentration of hFABP from baseline to 24 h after surgery (Con group: T1* versus* T0 *P* < 0.001, T2* versus* T0 *P* < 0.001, T3* versus* T0 *P* = 0.002, T4* versus* T0 *P* < 0.001, T5* versus* T0 *P* < 0.001, and T6* versus* T0 *P* < 0.001; EA group: T1* versus* T0 *P* < 0.001 T2* versus* T0 *P* < 0.001, T3* versus* T0 *P* < 0.001, T4* versus* T0 *P* < 0.001, T5* versus* T0 *P* < 0.001, and T6* versus* T0 *P* < 0.001), such as cTnI, and reached peaked point at T2; there were no differences between the groups at T0, while, compared to control group, the serum concentration of hFABP from T2 to T6 was significantly less in EA group (EA group* versus* Con group: T2, *P* = 0.029; T3, *P* = 0.031; T4, *P* = 0.047; T5, *P* = 0.030; T6, *P* = 0.029).

## 4. Discussion

In this study, we have demonstrated that EA not only plays a role of cardioprotection including antiarrhythmia, attenuating myocardium injury on patients undergoing heart valve replacement, but also reduced sufentanil consumption, the occurrence of PONV, POCD in perioperation, and improved MMSE scores and QOR-9 scores compared to the control group. We yet found EA significantly shortened the time to extubation, the first time to flatus, first activity out of bed, and length of postop hospital stay.

EA at the heart meridian acupoints has been reported to attenuate cardiac injury, such as decreasing myocardial enzymes and infarction [6, 7, 16, 17], and correcting arrhythmia [8, 19], which were induced by acute myocardial ischemia and reperfusion. It is well known that serum cTnI concentration was adopted as a sensitive biomarker to evaluate the severity of myocardial injury. However, Researchers supported hFABP, the sensitivity of the new assay was 98.25%, and specificity was 100% using the Randox kit as the reference kit [26]. Evidence proved that hFABP is superior to cTnI, CK-MB, and myoglobin for predicting all-cause mortality up to 5 years after CABG surgery and is released earlier and in larger amounts into the circulation because of myocardial injury [13]. Hasegawa et al. measured serial hFABP levels at 0, 1, 2, 3, and 6 hours after aortic unclamping in pediatric cardiac surgery; they and others demonstrated that the initial rapid increase and peak of hFABP approximately 1 hour after aortic unclamping and were followed by a fast decrease to within 10% of baseline by 24 hours [27, 28]. hFABP is highly sensitive and specific for predicting the outcomes of acute MI and coronary syndromes, often exceeding the current clinical “gold standard” of diagnosis-cTnI. Therefore, we detected the serum concentrations of the two biomakers with cTnI and hFABP. The data in our study presented that cTnI was significantly increased at the start of surgery and was markedly lowered in EA group compared to control group at the time points of 30 min, 60 min, 90 min, 360 min, and 1440 min postoperatively that differenced from the previous studies, which showed a significance at the time points of 6 h, 12 h, and 24 h postoperatively [16, 17]. In contrast, hFABP exerted the peak of hFABP approximately fifteen doubles over the baseline at 30 min after CPB (63.0 ± 4.8 versus 62.6 ± 4.8) and were followed by a fast decrease to three doubles of baseline at 24 hour after operation; these data indicate the differences from previous studies, but the serum concentrations of hFABP rising up were significantly slower in EA group as graphed in Figure 5 from the start of surgery to 30 minutes of CPB (*P* < 0.05) and were lower to control group at different time points postoperatively, which implied that EA could accelerate the serum concentrations of hFABP returning to close to the baseline. It might be a more specific and sensitive than cTnI as a biomarker of myocardial injury. All of these interpreted that EA reduced the myocardial injury.

The previous study showed that the cardiac arrhythmias induced by myocardial ischemia and reperfusion were attenuated by the pretreatment of acupuncture. In a randomized study, Gao and colleagues examined the antiarrhythmic effect of acupuncture in the rats subjected to simulative ischemia and reperfusion (SGIR) that might be due to inhibition of calcium overload, reduction of nonphosphorylated Cx43 which may cause cardiac electrically conductive disorder and result in arrhythmias eventually [19]. A study by 80 patients with persistent atrial fibrillation (AF) after restoring sinus rhythm with electrical cardioversion acupuncture treatment showed that acupuncture prevents arrhythmic recurrences after cardioversion and is reduced by 19%~24% for 12-month follow-up [8]. In addition, one case report which presented acupuncture significantly decreased the mean number of ventricular premature complexes (VPC) on a 36-year-old male chimpanzee (Pan troglodytes) diagnosed with frequent (VPC) [29]. Though the total rate of arrhythmia in our trial suggested that they were in common, importantly, EA significantly reduced the incidence of RVA and decreased the antiarrhythmic score, indicating that EA has a positive effect of antiarrhythmia.

Meanwhile, evidence shows that EA not only plays a cardioprotection but also significantly promotes recovery of neurological function and thus improves their quality of life [6, 7, 12, 16, 26, 30–32]. Gao and others observed that the old patients received EA at Baihui (DU20), Hegu (LI4), Neiguan (PC6), and Zusanli (ST36) 30 min before anesthesia induction to the end of operation assistant general anesthesia with noncardiac surgery on postoperative cognitive dysfunction (POCD), finding that EA reduced the occurrence of POCD (23.3% versus 46.7%) in aged patients and increased the scores of MMSE on the 2nd and the 4th day after surgery [14]. Some studies reported that electroacupuncture could optimize cognitive function undergoing abdominal operation or others [12, 33]. In our trial, patients with EA had a better cognitive significantly noting by the MMSE scores on the first day after surgery and reduced the incidence of POCD at the early days.

According to the theory of traditional Chinese medicine (TCM), performing surgery breaks the balanced state of the human body and disturbs the movement of both qi and blood, one of PC6's main functions is to regulate the function of the stomach to avoid the adverse flow of qi, and it is an effective acupoint for preventing nausea and vomiting. Thus PONV is on the list of medical conditions that may benefit from treatment with acupuncture, issued by the World Health Organization [34]. Our trial proved that EA shortened the time to flatus postoperatively, indicating that electroacupuncture accelerated the return of bowl function. The incidence of PONV in this study is revealed to be significantly decreased; the reason is not only due to the effect of electroacupuncture stimulated but also due to the reduced intraoperative opioid consumption, and thus we consider both of them contributed to the reduction occurrence of PONV.

As we know, pain is among the specific conditions for which acupuncture's efficacy is well accepted, according to a statement issued by the NIH. The antianalgesia effect of acupuncture is mediated mainly by activation of the descending inhibition system, including opioidergic endorphins, enkephalins, and dynorphin, adrenergic, and serotonergic pathways in both the central and peripheral nervous systems [11, 31, 35–37]. In major procedures, such as open heart surgery under cardiopulmonary bypass, patients in the combined acupuncture-drug group required only 13% of the total dose of fentanyl required in the general anesthesia group [38]. A review by Sun and colleagues, which included fifteen RCTs conducted up to 2008, revealed a 21–29% reduction in opioid consumption, lower incidence of opioid-related side effects such as nausea, dizziness, urinary retention, and decreased VAS score after acupuncture, which is generally considered to be clinically significant [39]. A study by Yu and colleagues also presented that acupuncture significantly reduced the injection dosage of morphine and enhanced the analgesia satisfaction rate in patients with cardiac surgery [15]. Our study found that the dosage of sufentanil was decreased by 18.8% approximately and the occurrence of rescue analgesia was not increased after surgery, which was similar to previous studies.

Interestingly, a study about application of acupuncture for open heart surgery suggested that acupuncture could shorten the patients of ICU stay by 15 hours and postoperative hospital stay by 3 days [38], and Yang and colleagues also got the same results [6]. In our study, the length of ICU stay was less in EA group by only 5 hours; we consider that this might be due to patients recruited who were not enough or the effects of administration in ICU. Otherwise, we found that patients in EA group were discharged from hospital earlier for 2 days about and that was similar to previous study. Finally, we found that QoR-9 and MMSE scores were significantly higher in EA group than control group for the first three days and felt more comfortable than that without EA. All above were consistent with ERAS: improving patient satisfaction and reducing morbidity and length of hospital stay.

The session of EA stimulus at bilateral PC6, PC4, GV24, and GV20 acupoints was twenty minutes before anesthesia induction to the end of surgery in our study; we have found significant results. We surmise that acupuncture is administered before operation; there will be better effect than if the acupuncture treatment is given during operation. Previous studies have demonstrated that acupuncture may be used to precondition motivation as well as functions relating to well-being and allostasis if it is administered before treatment program; the authors recommended that acupuncture should preferably be applied before the start of (not during) any or possibly all specific treatments to enhance the specific and nonspecific effects [40, 41].

*Strengths and Limitations*. In our present study, the patients were not informed of the allocation, and both groups were anesthetized equally; the acupuncturist conducting EA was masked to treatment assignment; none participated in data acquisition and analysis. Thus, our study's strengths include adequate blinding, standardized EA, and anesthesia protocols. However, we recognize the limitations of our present trial, one possible limitation of the present study was that the recruited patients had relatively normal left ventricle function, though there have been some reports of myocardial protection strategies with heart failure [32], whether this technique would be beneficial or harmful to the more severely dysfunctional heart remains to be investigated; the other possible limitation was that the sample size in this study was relatively small and patients collected are from a single centre. A larger sample size and large-scale multicentre clinical trials are still needed.

## 5. Conclusions

Overall, the findings from this trial suggest that EA plays an important role of cardioprotective effect and is useful in reducing the occurrence of complications and also in improving the quality of life in patients after heart valve replacement with CPB at the early days after operation which potentially benefits patients to recovery after surgery.

## Figures and Tables

**Table 1:** Arrhythmia scoring system.

Arrhythmia score	Type of arrhythmia
0	No arrhythmia
1	Atrial arrhythmias or occasional PVC
2	Frequent PVC
3	VT (1-2 episodes)
4	VT (>3 episodes) or VF (1-2 episodes)

**Table 2:** Characteristics and surgical details of study population.

	Control group	EA group	*P* value
	*n* = 20	*n* = 20	
Gender (male/female)	9/11	8/12	NS
Age in years	47 ± 5	44 ± 6	NS
Weight (kg)	59 ± 6	58 ± 9	NS
LVEF (%)	57 ± 7	59 ± 5	NS
Heart function degree (II/III)	4/16	3/17	NS
Rhythm of heart before surgery (sinus/atrial fibrillation)	8/12	8/12	NS
Valve replacement			NS
Mitral valve	12	13	
Aortic valve	2	3	
Double^a^ valve	6	4	
Anesthesia time (min)	308 ± 38	304 ± 45	NS
Operation time (min)	207 ± 32	206 ± 49	NS
CPB time (min)	91 ± 39	87 ± 29	NS
Aorta cross-clamp time (min)	66 ± 25	68 ± 31	NS
Total volume (ml)	2264 ± 462	2438 ± 636	NS
Anesthetic			
Midazolam (mg)	16 ± 2	15 ± 3	NS
Vecuronium (mg)	16 ± 2	16 ± 3	NS
Sufentanil (*μ*g)	355 ± 39	288 ± 76	0.002
Propofol (mg)	822 ± 169	737 ± 253	NS

LVEF = left ventricular ejection fraction. ^a^Mitral and aortic. CPB = cardiopulmonary bypass time. NS = no significant.

**Table 3:** Postoperation data of study population.

	Control group	EA group	*P* value
	(*n* = 20)	(*n* = 20)	
Time to extubation, min	548 ± 186	430 ± 171	0.045
ICU LOS, hours	18.3 ± 1.7	17.0 ± 2.7	NS
First flatus time, hours	32.7 ± 11.4	20.6 ± 7.5	0.000
First out of bed activity, hours	101.9 ± 12.7	96.8 ± 11.3	0.026
Occurrence of PONV (%)	35	10	0.000
Rate of rescue analgesia^a^ (%)	35	25	NS
Occurrence of radiation^a^ (%)	15	5	0.032
Occurrence of POCD^a^ (%)	50	15	0.000
Postop hospital LOS, day	15.5 ± 1.9	13.0 ± 4.0	0.018

^a^Occurrence assessment was in the first three days after operation; LOS = length of stay; NS = no significant.

**Table 4:** Antiarrhythmia of electroacupuncture.

	Control group	Electroacupuncture group	*P* value
	(*n* = 20)	(*n* = 20)	
Type of heartbeat restored after aorta cross-clamp removal (%)			NS
Spontaneous	80	90	
Defibrillation	20	10	
Rate of arrhythmic, %			
Total occurrence	55	55	NS
RVA in the first three days	45	25	0.005
Arrhythmia score	1.8 ± 0.5	1.2 ± 0.3	0.014

RVA = rapid ventricular arrhythmias; NS = no significant.
